# Deep Vision for Breast Cancer Classification and Segmentation

**DOI:** 10.3390/cancers13215384

**Published:** 2021-10-27

**Authors:** Lawrence Fulton, Alex McLeod, Diane Dolezel, Nathaniel Bastian, Christopher P. Fulton

**Affiliations:** 1 College of Health Professions, Texas State University, San Marcos, TX 78666, USA; 2 McCoy College of Business, Texas State University, San Marcos, TX 78666, USA; am@txstate.edu; 3 Health Information Management, College of Health Professions, Texas State University, Round Rock, TX 78665, USA; dd30@txstate.edu; 4 United States Military Academy, West Point, NY 10996, USA; nathaniel.bastian@westpoint.edu; 5 United States Air Force, Palmdale, CA 93551, USA; christopher.fulton.6@us.af.mil

**Keywords:** deep vision, breast cancer, machine learning, region of interest detection

## Abstract

**Simple Summary:**

Breast cancer misdiagnoses increase individual and system stressors as well as costs and result in increased morbidity and mortality. Digital mammography studies are typically about 80% sensitive and 90% specific. Improvement in classification of breast cancer imagery is possible using deep vision methods, and these methods may be further used to identify autonomously regions of interest most closely associated with anomalies to support clinician analysis. This research explores deep vision techniques for improving mammography classification and for identifying associated regions of interest. The findings from this research contribute to the future of automated assistive diagnoses of breast cancer and the isolation of regions of interest.

**Abstract:**

(1) Background: Female breast cancer diagnoses odds have increased from 11:1 in 1975 to 8:1 today. Mammography false positive rates (FPR) are associated with overdiagnoses and overtreatment, while false negative rates (FNR) increase morbidity and mortality. (2) Methods: Deep vision supervised learning classifies 299 × 299 pixel de-noised mammography images as negative or non-negative using models built on 55,890 pre-processed training images and applied to 15,364 unseen test images. A small image representation from the fitted training model is returned to evaluate the portion of the loss function gradient with respect to the image that maximizes the classification probability. This gradient is then re-mapped back to the original images, highlighting the areas of the original image that are most influential for classification (perhaps masses or boundary areas). (3) Results: initial classification results were 97% accurate, 99% specific, and 83% sensitive. Gradient techniques for unsupervised region of interest mapping identified areas most associated with the classification results clearly on positive mammograms and might be used to support clinician analysis. (4) Conclusions: deep vision techniques hold promise for addressing the overdiagnoses and treatment, underdiagnoses, and automated region of interest identification on mammography.

## 1. Introduction

An estimated 2.3 million women were diagnosed with breast cancer globally in 2020, and female breast cancer has surpassed lung cancer as the most diagnosed cancer in the world [1]. The odds of a female being diagnosed with breast cancer have increased from 11:1 in 1975 to 8:1 today [2]. Globally, breast cancer is the most prevalent type of cancer with the most disability-adjusted life years [3]. Age-adjusted rates show growth in breast cancer diagnoses of 0.3% per year [4,5].

In the United States, about 13% of women will be diagnosed with breast cancer over their lifetimes [6]. Although breast cancer fatality rates have declined 1% since 2013, likely due to advancements in treatment, it is still the second most fatal cancer diagnosis [6]. Fatality rates from breast cancer decreased 7% from 2002 to 2003 (possibly due to an evidence-based reduction in the use of hormone replacement therapy [6,7]). However, the increased use of breast implants (48% increase from 2000 to 2018, the pre-COVID-19 era [8]) is linked to an increased risk of breast implant associated anaplastic large cell lymphomas (BIA-ALCL). These lymphomas are rare and difficult to detect [9], facts associated with a reduced probability of 5-year survival. The Scientific Committee on Health, Environment, and Emerging risks considers there to be moderate evidence of a causal link between textured breast implants and BIA-ALCL [10].

Mammography reduces breast cancer mortality and is thus an important diagnostic tool [11,12,13]. The implementation of breast cancer screening has resulted in the increase in ductal carcinoma in situ, a pre-invasive form of cancer that may or may not progress [14]. Misdiagnoses of mammography results in increased morbidity, mortality, stress, and costs. False positive rates (FPR), estimated in one study to be 121.2 per 1000 or 12.12% [15], are associated with overdiagnoses and overtreatment and must be addressed while simultaneously improving false negative rates (FNR) to reduce morbidity and mortality. One study estimates that sensitivity and specificity of digital mammography studies is 80% and 90%, respectively [16].

The use of human–computer interaction in mammography studies is becoming increasingly important [17]. Advances in machine learning (ML) have improved both positive predictive validity (PPV) and negative predictive validity (NPV) in breast cancer identification. Application of fast opposite learning weights, an ML technique that may improve classification performance, resulted in FNR of 9.9% and FPR of 11.94% in one study [18]. A study by Ertosun and Rubin used Convolutional Neural Network (ConvNet) achieved 0.78 precision in mammography classification [19]. Muramatsu et al. achieved 84% accuracy using ConvNet. ML techniques hold promise for improving PPV and NPV [20]. Salama and Aly were able to achieve impressive classification metrics (98% accuracy, 98% specificity, and 98% sensitivity) on small samples with augmented imagery [21].

Image segmentation attempts to divide an image into non-overlapping areas. Techniques that have been applied to this task include thresholding, watershed-based methods, graph-based methods, clustering, and region-based approaches [22]. Thresholding methods are often exceedingly basic, seeking to enhance grayscale images based on intensity values [23]. Smooth boundaries and unimodal histograms (pixel value) pose problems for this method [22]. Watershed-based methods consider the image is a contour map and seek to find the lowest/highest points [24] but are sensitive to noise and over-segmentation and are computationally expensive as gradient calculation is required [22]. Graph-based methods subset images using nodes and edges where the edges never overlap and have been used for region of interest (ROI) identification in breast cancer [25]. They are, again, computationally problematic for larger image sizes [26]. Clustering methods (K-means, hierarchical, etc.) seek to group like pixels together in order. These algorithms are sensitive to outliers, noises, and initial values [25]. Region-based methods seek to divide an image into homogenous regions but are sensitive to the initial region selected as the ‘seed’ [22].

Recent efforts have sought to use the output of a ConvNet classification model to identify ROIs on photographs without knowing where the actual lesion may exist. Techniques such as ‘guided backpropagation’ [27] and ‘deconv’ [28] have been used to identify image areas that activate the neurons of ML models. The gradient of the loss function with respect to an image representation (convolutional layer) can be used to pinpoint tissue segments that might be of concern even if the initial read of that study was negative without previously classified imagery (unsupervised learning). Recent efforts have shown that such methods are highly capable of producing bounding boxes for imagery [29]. It is therefore possible to identify regions of interest (ROIs) in medical imagery by simply ‘mining’ the layers of a ConvNet classification algorithm without additional segmentation analysis.

This study addresses the problems of overdiagnoses and overtreatment, as well as underdiagnoses with its associated increases in morbidity and mortality by (1) improving classification of mammography using supervised learning (learning from images where the classification label is in the data) and (2) implementing guided backpropagation to paint gradient contours of the ROIs using unsupervised learning. This research supports efforts to reduce morbidity and mortality while simultaneously addressing overdiagnosis and overtreatment.

## 2. Materials and Methods

### 2.1. Data, Software, and Hardware

Data are publicly available from the Digital Database for Screening Mammography (DDSM) [30] and the Curated Breast Cancer Imaging Subset of DDSM [31] and provided by Google’s Kaggle.com, accessed on 5 January 2021 [32]. This analysis is a precursor to follow-on work supported by a National Cancer Institute Data Transfer Use Agreement (PLCOI-742). The data consisted of 71,249 images and labels, 55,885 pre-designated for training and 15,364 reserved for testing. The image data were sized 299 × 299 (single channel grayscale) but were augmented to three-color (‘RGB’) for use in models by replicating the channel. The label data included dichotomous classification (0 for true negative, 1 otherwise). All analyses were performed in Anaconda Python 3.7 and are available on Github [33]. An in-kind high performance computing grant from Advanced Micro Devices (250 teraflops computing power) provided the computational power for model training. The observations (pixels) used for the image data only were 299 pixels × 299 pixels × 3 channels × 71,249 images = 19.1 billion pixels.

### 2.2. Training, Validation, and Test Sets

The training set was further randomly subdivided into training and validation sets (80% and 20%, respectively) resulting in the final data sets of size 44,708 (training), 11,177 (validation), and 15,364 (test). By further splitting the training data, machine learning algorithms use the retained training data to estimate model performance on the validation set prior to estimation of the test set as a mechanism for preventing overfitting and to compare model hyperparameters. The final tuned model used both the training and validation sets to predict the pristine test set.

### 2.3. Image and Label Preprocessing

Each of the image intensity values were scaled between 0 and 1 (min-max scaling). Test data remained untransformed other than pixel scaling. Label data were complete.

### 2.4. Architecture

**Figure 1:**
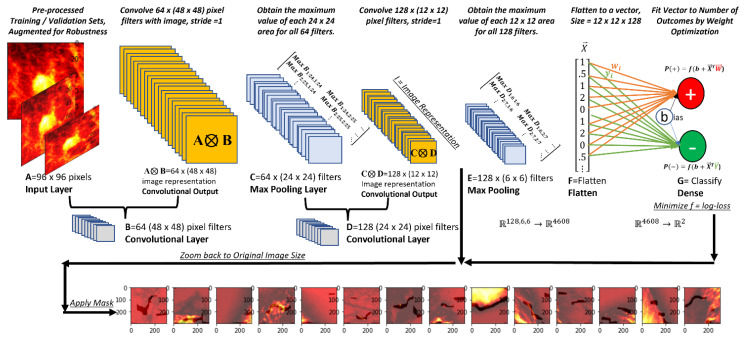
ConvNet architecture and graphical research overview.

ConvNet models were built on the training images and performance calculated using the validation set. Then the models were used to classify the unseen but labeled test set. ConvNet is an ML network architecture capable of taking radiological images and making outcome predictions. ConvNets are often used for image denoising [34], as they preserve the important spatial relationships and features. Figure 1 is a basic ConvNet architecture.

### 2.5. Deep Vision Basics

An image is represented as an x-y mapping of pixel color intensity, z. For color images, there are three matrices that form an array, one each for red, blue, and green intensities. One could consider ConvNet to be a dimension reduction protocol that reduces the image size while retaining important features and spatial information. The reduced image representations are eventually flattened, where each pixel is then part of a variable vector that might be used to forecast class membership by minimizing a log-loss or ‘softmax’ function, somewhat analogous to logistic regression for the two-class cases and multinomial regression for the multi-class cases.

### 2.6. Supervised Classification

Assume that we are evaluating pre-processed images of 96 × 96 pixels with 1 color channel (‘A’ in Figure 1). Many image filters of smaller size (e.g., 48 × 48 pixels, ‘B’ in Figure 1) are passed over the original image, section by section, one or more pixels at a time. The tensor product, A⦻B, is taken for each of these regions (convolutional output). Moving each of our 48 × 48 filters over the original 96 × 96 images 1 pixel at a time generates a new 48 × 48 matrix. The convolutional layer output, a set of smaller image representations, are additionally processed through a function for classification improvement (a rectifier function that adds additional nonlinearity to images, f (x) = max (0, x). Smaller filters (24 × 24 pixels in Figure 1, ‘C’) are applied to convolutional layers. These filters execute ‘maximum pooling’ (keeping the maximum value in each region) or ‘average pooling’ (averaging all values). Multiple layers (‘D’ and ‘E’ in Figure 1) are then combined in a network structure. The final image representation is flattened into a single vector (dense layer, ‘F’ in Figure 1). The inner product of this vector and weights (tuned through nonlinear optimization) are added to a constant (bias) and processed through a ‘softmax’ function (‘G’ in Figure 1). The softmax function provides the probability of negative (−) or non-negative (+) imagery Equation (1).

(1) $$P\left(+\right)=\frac{e^{-X^{T}W}}{e^{-X^{T}W}+e^{-X^{T}Y}},\;\;P\left(-\right)=\frac{e^{-X^{T}Y}}{e^{-X^{T}W}+e^{-X^{T}Y}}.$$

Equation (1) estimates the probability that an image will belong in either the negative (−) or non-negative (+) class. In this equation, *X^T^* is the flattened vector produced by the ConvNet (‘F’ in Figure 1), and the weights for negative (−) and non-negative (+) conditions are *W* and *Y*, respectively. The probability for each class is estimated by dividing the exponent of the weighted vector for each class by the sum of the weighted vectors for both classes.

For two-category classification, only one equation rather than two is needed. A sigmoid or log-loss equation classifies either negative (−) or non-negative (+) studies Equation (2).

(2) $$P\left(+\right)=\sigma \left(x\right)=\frac{1}{1+e^{-x}}$$

In Equation (2), we define σ(x) as the sigmoid function which takes the vector data from the flattened layer and estimates the probability of non-negative (+) results.

ConvNets require tuning of the weights and filter values through nonlinear minimization of a loss function, typically the log-loss (binary cross-entropy, Equation (3)).

(3) $$L=-\frac{1}{N}\overset{N}{\underset{i=1}{\sum }}y_{i}log\left(p_{i}\right)+(1-y_{i})log\left(1-p_{i}\right)$$

In Equation (3), *L* is the loss function, *N* is the number of items classified, *y* is the true class membership (either negative or non-negative), and *p* is the probability estimate for the group membership. A correctly classified observation (e.g., a true ‘1’ predicted to be such with probability 0.999) results in a value near 0. An incorrectly classified observation (e.g., a true ‘1’ predicted to be ‘0’ with 0.999 probability) results in a value of 4.605. Thus, minimizing binary cross-entropy through nonlinear optimization attempts to improve the classification.

### 2.7. Specific Architecture, Classification Problem

For this analysis, we evaluated several pre-existing architectures that have shown to perform well on the image classification task. The best-performing architecture of those considered is presented here and is based on the Visual Geometry Group (VGG) of Oxford [35]. The architecture, known as VGG-16, includes 13 convolutional layers and 5 maximum pooling layers. Global average pooling was added to this baseline architecture to further reduce dimensionality, and a single neuron with sigmoid activation function provided the final estimate of the probability of a non-negative result. While we started with pre-trained weights for the VGG-16 architecture, we allowed these to be updated during nonlinear optimization.

Images were batched in groups of fed into the architecture. Validation metrics were estimated, and hyperparameter tuning was performed. The final selected image batch size was 32, and the chosen nonlinear optimizer was an adaptive gradient algorithm known as ‘AdaGrad’ [36]. A total of 50 epochs were set with early stopping conditions (which activated after epoch 25).

### 2.8. Unsupervised Region of Interest (ROI) Identification

Traditional radiological imagery is often devoid of ROI segmentation, which depicts the location of anomalies. Without previously identified ROIs (as is the case with the imagery in this study), it is not possible to train supervised models to isolate ROIs. It is, however, possible to return a small image representation from the closest convolutional layer of a model, evaluate the gradient of the loss function with respect to the image at that layer using computational graphs, find the portion of the gradient that maximizes the classification probability, and re-map that gradient location back to the original image. In doing so, the areas of the original image that are most highly influential for the classification model can be found. Further, an image contour overlay can be built to identify the areas most associated with the classification determination based on important values of the gradient of the loss function with respect to the image. In this study, we avoid guided backpropagation as well as other non-gradient based methods based on recent experimental findings [37].

**Figure 2:**
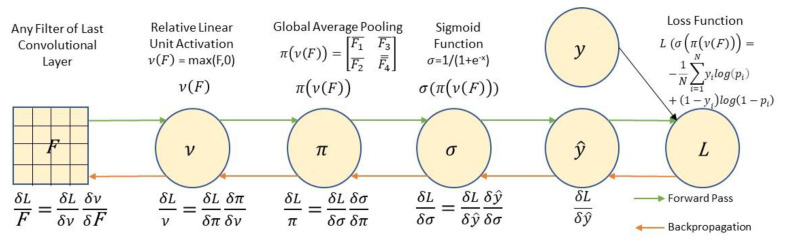
Backpropagation to find $\frac{\delta L}{F}$.

Figure 2 represents the last functions of the ConvNet provided the probability prediction for the image. For each filter F in the last convolutional layer, we seek the gradient of the loss function with respect to the image, $\frac{\delta L}{F}$. The forward pass (green arrows) results in a composite function from the filters through the rectifier, global average pooling, the sigmoid function to the forecast. To calculate $\frac{\delta L}{F}$ requires that we ‘backpropagate’ the gradients (red arrows) using the chain rule until we are able to estimate our loss gradient with respect to the filter. These calculations are supported by TensorFlow and need not be done manually [38].

.

## 3. Results

### 3.1. Descriptive Statistics

The data were imbalanced, as 86.96% of the images were negative. ConvNets are robust to imbalanced data, so addressing this imbalance by over or under sampling (for example) was unnecessary. We investigated image augmentation using 10 degrees of random rotation, 20% zoom in and out, 10% height and width shifts, 10% shearing, and horizontal flipping, as augmentation sometimes produces more robust classifiers [39]. We saw no improvement with image augmentation possibly due to issues with local optima and thus proceeded with the original images.

**Figure 3:**
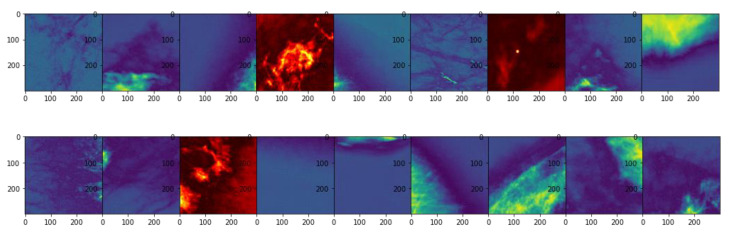
Image plots of slides with non-negative results depicted in red.

Images were saved as three channel (‘RGB’) color images to support the baseline VGG-16 architecture. Several of the images were plotted using various color enhancements to verify data quality (see Figure 3 for an example). An autoencoder to reduce image noise was implemented but added no observable benefit to the classification.

### 3.2. Classification Results

The VGG-16 architecture resulted in 98% classification accuracy, 83% sensitivity (recall of positives), and 99% specificity (recall of negatives) on the pristine test set after 25 epochs. These metrics are all improved over previous studies including [19,20]. The positive predictive value (precision for positive cases) is 0.93, indicating that 93% of the patients informed of positive study results were true positives, much better than [19]. Table 1 provides the complete results of predicting the test result with the trained model, while Table 2 is the confusion matrix (the classification matrix with incorrect and correct predictions labeled).

Table 1 shows that the classification process worked well. The high recall (99%) on the negative patients suggests that overdiagnosis and overtreatment would be reduced. The sensitivity of 83% might even be improved with additional a priori manipulation as well as larger datasets.

In Table 2, the FPR is (131/1801) = 7.3% while the FNR is (334/13,563) = 2.5%. These metrics are much better than those seen in [18].

### 3.3. Unsupervised Gradient Mapping

**Figure 4:**
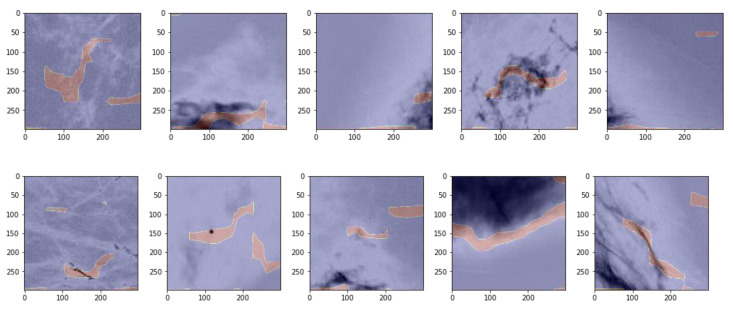
The first 10 positive images in the data set with the gradient loss contour mapped.

We used TensorFlow’s ‘GradientTape’ function to calculate the gradient of the loss function at the last convolutional layer’s reconstructed image, which includes 512 filters of size 18 × 18 pixels. After producing the gradient map, we zoomed the image back to 299 × 299 to produce contour overlays for the images. To further identify areas of concern, we filtered for the top 10% of the image values. In Figure 4, we show the first 10 positive images in the data set in gray scale with the contour mask of the gradient superimposed (showing as pink).

Figure 4 shows that the unsupervised classification is able to pick up features in the images associated with anomalies. In Row 1, plots 2 through 5 and in Row 2 plots 3 through 5, it is clear that the gradient of the loss with respect to the image considers the darkish areas to be associated with anomalies. In Row 2, plots 1 and 2 appear to identify a high density ‘line’ and ‘point’, respectively. The contour plots appear to capture the boundaries of interesting tissue in the plots, which is to be expected, as the maximum values of the gradient of the loss with respect to the image representation are those areas most closely associated with the algorithm’s classification decision.

## 4. Discussion

Supervised classification of mammography detection is effective in achieving high sensitivity, specificity, precision, and overall accuracy. The model presented here was highly specific (99%) with reasonable sensitivity (83%). This particular model would effectively address the problems of overdiagnosis and overtreatment, and slightly address the problems of morbidity and mortality associated with breast cancer. Refinements to the architecture, weights, and other elements of the model would be expected to improve its performance; however, a relatively common ConvNet architecture was capable of achieving these results on a sufficiently large dataset with reasonably sized images without significant hyperparameter tuning or image manipulation.

Backpropagation techniques worked well to produce contour maps for the loss gradient with respect to the image representation. Zooming these contour maps from the last convolutional layer back to the original image size provided reasonable visual representation of the areas that are (by definition) most closely associated with the classification process. Thus, it is relatively straightforward to determine regions of interest during the ML classification process without running separate models. Providing pre-marked images to physicians, particularly when ML models and physicians have different ‘opinions’ regarding the mammography, is an important consideration for improving FPR and FNR rates.

A limitation of this study is that it uses previously curated images of fixed and relatively small size. Larger files (e.g., Aperio ScanScope.svs image sets) require significantly more preprocessing and computational power. Further, the positive mammography lacks confirmed ROIs, so the contour masks may not be interpreted alone. In addition, this study focused on the classification of non-negative imagery. Classification using the BI-RADS classification scheme is also possible but beyond the scope of this initial study.

Future work involving imagery obtained by the National Cancer Institute under a Data Transfer Agreement (PLCOI 742) is ongoing. This work requires significant processing power from a cloud-based high performance computing cluster supported by Advanced Micro Devices under an in-kind grant.

## 5. Conclusions

Deep vision holds much promise for breast cancer classification, as it is able to find relationships among spatially related pixels that the human eye cannot detect. Thus, it is possible for deep vision to detect cancers missed by clinical professionals under even optimal conditions assuming that properly classified training data are available from which the algorithms can learn.

ML techniques in general hold great promise for both the supervised classification and unsupervised segmentation problems. These techniques are likely to assist radiologists and clinicians in reducing FNR and FPR rates, addressing the issues of overdiagnosis and overtreatment while simultaneously reducing morbidity and mortality. It is clear that the role of the ML in assisting clinicians with diagnoses and ROI identification will increase given its demonstrated potential.

## Figures and Tables

**Table 1:** Metrics for predicted test set data, 97% accuracy.

Metric	Size	Precision	Recall	F1-Score
Negative	13,360	0.98 ^0^	0.99 *	0.98 ***
Positive	2004	0.93 ^1^	0.83 **	0.88 ***
Weighted Average	15,364	0.97	0.97	0.97

^0^ Negative Predictive Value, ^1^ Positive Predictive Value, * Sensitivity, ** Specificity, *** Harmonic Mean of Precision & Recall.

**Table 2:** Confusion matrix for the classification problem.

Actual/Prediction	Negative Prediction	Positive Prediction	Total
Negative	13,229	131	13,360
Positive	334	1670	2004
Total	13,563	1801	15,364

## Data Availability

Data are available online with a free account. The address for access follows: https://www.kaggle.com/skooch/ddsm-mammography, accessed on 5 January 2021.
